# The effect of smoking on MRI lesion resolution in NMOSD-AQP4 and MOGAD

**DOI:** 10.1177/13524585231188485

**Published:** 2023-08-01

**Authors:** David Berhanu, Miguel Leal Rato, Silvia Messina, Maria Isabel Leite, Ruth Geraldes, Jacqueline Palace

**Affiliations:** Nuffield Department of Clinical Neurosciences, University of Oxford, Oxford, UK; Serviço de Imagiologia Neurológica, Centro Hospitalar Universitário Lisboa Norte, EPE, Lisboa, Portugal; Nuffield Department of Clinical Neurosciences, University of Oxford, Oxford, UK; Serviço de Imagiologia Neurológica, Centro Hospitalar Universitário Lisboa Norte, EPE, Lisboa, Portugal; Nuffield Department of Clinical Neurosciences, University of Oxford, Oxford, UK; Department of Neurology, Frimley Health NHS Foundation Trust, Frimley, UK; Nuffield Department of Clinical Neurosciences, University of Oxford, Oxford, UK; Department of Clinical Neurology, John Radcliffe Hospital, Oxford University Hospitals Trust, Oxford, UK; Nuffield Department of Clinical Neurosciences, University of Oxford, Oxford, UK; Department of Neurology, Frimley Health NHS Foundation Trust, Frimley, UK; Nuffield Department of Clinical Neurosciences, University of Oxford, Oxford, UK; Department of Clinical Neurology, John Radcliffe Hospital, Oxford University Hospitals Trust, Oxford, UK

**Keywords:** Lesion resolution, smoking, MRI, NMOSD, AQP4, MOGAD, inflammation

## Abstract

**Background:**

The effect of smoking on the resolution of magnetic resonance imaging (MRI) lesions in patients with neuromyelitis optica spectrum disorders with aquaporin-4 positive antibody (NMOSD-AQP4) and myelin oligodendrocyte glycoprotein antibody-associated disease (MOGAD) has not been studied before.

**Objective:**

We aimed to determine the effect of smoking on lesion resolution in MRI and assess its correlation with clinical recovery after a relapse.

**Methods:**

We conducted a cohort study including NMOSD-AQP4 and MOGAD patients with acute and follow-up MRI scans. We collected demographic, clinical, imaging and smoking data. Logistic regression models were fitted to predict the effect of smoking on lesion resolution and to assess whether clinical recovery was associated with MRI lesion resolution.

**Results:**

A total of 105 patients were included (57 with NMOSD-AQP4 and 48 with MOGAD). Current and past smoking was associated with a higher risk of persistent lesions in NMOSD-AQP4 and MOGAD (risk ratio (RR) = 3.4, 95% confidence interval (CI) = 2.5–4.7, *p* < 0.001). Additionally, the presence of lesion resolution was associated with better clinical recovery (RR = 1.9, 95% CI = 1.7–2.2, *p* < 0.001).

**Conclusion:**

Smoking is associated with worse MRI lesion resolution in patients with NMOSD-AQP4 and MOGAD, and lesion resolution correlates with clinical recovery. Our findings suggest a detrimental effect of smoking in inflammatory central nervous system (CNS) diseases.

## Introduction

Neuromyelitis optica spectrum disorder with aquaporin-4 positive antibody (NMOSD-AQP4) and myelin oligodendrocyte glycoprotein antibody-associated disease (MOGAD) are a group of antibody-associated inflammatory diseases with overlapping features, where disability occurs as a relapse-associated worsening. However, disease course, clinical relapse recovery and lesion evolution differ between these disorders, which may be linked to differences in the resolution of relapse-associated inflammation profile.^1–5^ Overall, little is known about what factors and comorbidities influence magnetic resonance imaging (MRI) lesion resolution and clinical recovery in these patients.

Vascular risk factors have a known detrimental effect in inflammatory diseases of the central nervous system (CNS) such as MS, with smoking in particular being associated with greater disability and a higher burden of MRI lesions.^6–8^ In NMOSD, previous studies reported that smoking affects the severity of the disease^9^ and may be associated with poor relapse recovery in NMOSD-AQP4.^10^ However, the impact of smoking on MRI lesion resolution, and the role of MRI as a surrogate for clinical improvement, has not been studied before.

We aimed to determine the effect of smoking on MRI lesion resolution and the association of lesion resolution with clinical recovery in people with NMOSD-AQP4 and MOGAD.

## Material and methods

### Study design and participants

We conducted a retrospective cohort study of patients followed at the national commissioned service for NMOSD/MOGAD in Oxford. All patients fulfilling the NMOSD-AQP4 criteria^11^ or the MOGAD diagnostic criteria,^12^ who gave consent according to local ethic regulations, were included. Additional inclusion criteria comprised: (1) available details of smoking history and (2) available acute MRI scan within 6 weeks of a first relapse in a certain CNS region and follow-up MRI (⩾4 months after baseline) without a relapse in between. The main outcome was lesion resolution in MRI across smoking status. As a secondary endpoint, we evaluated the relationship between lesion resolution and clinical recovery.

### Clinical and imaging data

We retrieved demographic and clinical data, including age at time of relapse, sex, disease duration until relapse, follow-up time between MRI scans, smoking status (current-, past-, never-smoker), vascular risk factors (hypertension, dyslipidaemia and diabetes), diagnosis, relapse type/region (optic neuritis, transverse myelitis, brain attack), lesion location (supratentorial, infratentorial, spinal cord) and relapse recovery. Standard clinical MRI scans with varying scanner fields and protocols according to clinical practice, including T1 and T2 weighted sequences from the CNS region of relapse (brain, spinal cord or orbit), were used to assess lesion resolution.

Lesion resolution was scored as complete (no lesion remaining in follow-up imaging), partial (some improvement in number or size compared to baseline, but residual lesions observed) or absent (lesion persistence, no evidence of resolution, lesions overlapping with baseline scan) by an investigator (D.B.) with previous neuroradiological experience. Inter-rater variability was tested on 12.4% of randomly selected subset of patients by another rater (M.L.R.). Both raters were blinded for clinical data, including smoking status, at the time of MRI scoring.

Clinical recovery from relapse was scored by the patient’s clinician as complete (full recovery with improvement to baseline), partial (clinical improvement, not reaching previous function) or absent (no or mild improvement with poor residual outcome).

### Statistical analysis

Inter-rater agreement was measured by weighted Cohen’s kappa coefficient. We performed a multivariate log regression model to determine the effect of smoking on MRI lesion resolution. A modified Poisson approach with robust error variances was used to account for convergence and outcome prevalence.^13^ The outcome for the fitted model was the risk ratio (RR) of lesion persistence, obtained by converting lesion resolution into a binary outcome (combination of partial and complete resolution into a single category). We included diagnosis, age at relapse, sex, disease duration until relapse, region of relapse, CNS lesion location and follow-up time between imaging studies as possible confounders. We performed a post hoc analysis of the effect of lesion location on lesion resolution. Statistical significance was set at *p* < 0.05. All analyses were performed using Stata version 16.1 (StataCorp LLC, Texas).

## Results

Of the 638 eligible patients in the cohort, 105 patients met the inclusion criteria, comprising 57 NMOSD-AQP4 patients and 48 MOGAD patients (Supplemental Figure 1). Demographic, clinical and imagiological characteristics of the cohort according to smoking status are summarized in Table 1.

**Table 1.** Demographic and clinical characteristics of the full cohort.

	Total	Never-smoker	Past-smoker	Current-smoker	*p* value
	*n* = 105	*n* = 65	*n* = 26	*n* = 14	
Diagnosis					0.30
NMOSD-AQP4	57 (54%)	38 (58%)	14 (54%)	5 (36%)	
MOGAD	48 (46%)	27 (42%)	12 (46%)	9 (64%)	
Age at relapse, years	43 (18)	40 (19)	48 (14)	46 (14)	0.13
Female sex	71 (68%)	44 (68%)	18 (69%)	9 (64%)	0.95
Disease duration at relapse, years	0.1 [0.0–2.5]	0.1 [0.0–0.9]	0.2 [0.0–5.4]	0.5 [0.1–5.7]	0.09
Time between MRI scans, years	1.2 [0.5–2.2]	0.9 [0.5–1.9]	1.4 [0.6–3.8]	1.7 [0.6–2.6]	0.13
Region of relapse					0.59
Brain attack	27 (26%)	17 (26%)	6 (23%)	4 (28%)	
Myelitis	60 (57%)	40 (62%)	14 (54%)	6 (43%)	
Optic neuritis	18 (17%)	8 (12%)	5 (19%)	5 (36%)	
CNS lesion location					0.08
Supratentorial	30 (29%)	13 (20%)	9 (35%)	8 (57%)	
Infratentorial	18 (17%)	14 (22%)	3 (12%)	1 (7%)	
Spinal cord	57 (54%)	38 (58%)	14 (54%)	5 (36%)	
Clinical relapse recovery					<0.001
Absent	18 (17%)	1 (2%)	10 (38%)	7 (50%)	
Partial	48 (46%)	31 (48%)	10 (38%)	7 (50%)	
Complete	39 (37%)	33 (51%)	6 (23%)	0 (0%)	
Lesion resolution					<0.001
Absent	31 (30%)	2 (3%)	16 (62%)	13 (93%)	
Partial	33 (31%)	27 (42%)	5 (19%)	1 (7%)	
Complete	41 (39%)	36 (55%)	5 (19%)	0 (0%)	

NMOSD-AQP4: neuromyelitis optica spectrum disorder with aquaporin-4 positive antibody; MOGAD: myelin oligodendrocyte glycoprotein antibody-associated disease; MRI: magnetic resonance imaging; CNS: central nervous system; IQR: interquartile range; ANOVA: analysis of variance.

Data are presented as mean (SD), median [IQR] or *n* (%). *p* values are from one-way ANOVA, Kruskal–Wallis test, chi-square test and Fisher’s exact test as appropriate.

### Association of smoking status with MRI lesion resolution

**Figure 1.**
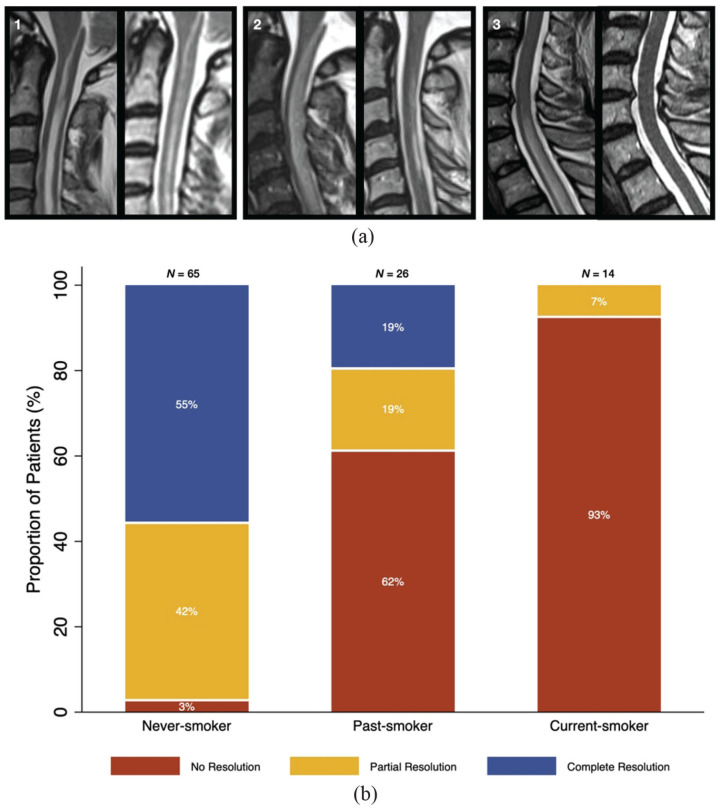
Association of MRI lesion resolution with smoking status in NMOSD-AQP4 and MOGAD. (a) Visual scoring of lesion resolution showing: (1) absent lesion resolution in a patient with NMOSD-AQP4 at 15-month follow-up MRI; (2) partial lesion resolution in a patient with NMOSD-AQP4 at 12-month follow-up MRI; (3) complete lesion resolution in a patient with MOGAD at 12-month follow-up MRI. (b) Association of MRI lesion resolution with smoking status. MOGAD: myelin oligodendrocyte glycoprotein antibody-associated disease; NMOSD-AQP4: neuromyelitis optica spectrum disorder with aquaporin-4 positive antibody.

There was an overall almost perfect inter-rater agreement (weighted κ = 0.81) in the subset of patients selected for imaging review (*n* = 13, 12.4%). Figure 1(a) shows an example of complete resolution, partial resolution and lesion persistence/absent resolution.

Lesion persistence was significantly more frequent in current-smokers (93%) than in past-smokers (62%), and in both smoker groups than in never-smokers (3%). Additionally, proportions of partial and complete resolution were higher in never-smokers and lower in current-smokers (Table 1, Figure 1(b)). None of the patients in the current-smoker group achieved complete lesion resolution on follow-up MRI. Overall, smoking was associated with a higher risk of persistent lesions in MRI (RR = 3.4, 95% confidence interval (CI) = 2.5–4.7, *p* < 0.001).

### Multivariate logistic model to predict lesion persistence

In a regression model to predict the association of lesion persistence with smoking and adjusting for predefined covariates, including diagnosis of NMOSD-AQP4 or MOGAD, the risk of lesion persistence in follow-up MRI was increased in current-smokers (RR = 26.6, 95% CI = 6.6–106.6, *p* < 0.001) and past-smokers (RR = 18.7, 95% CI = 4.7–74.6, *p* < 0.001) compared with never-smokers (Table 2). The diagnosis of NMOSD-AQP4 increased the risk of persistent lesions compared with the diagnosis of MOGAD (RR = 1.9, 95% CI = 1.2–3.2, *p* = 0.01), independently of smoking status, with patients with NMOSD-AQP4 having overall worse lesion resolution. Adjusting for other vascular risk factors did not impact the effect of smoking on lesion resolution, but due to small number of patients with available information on hypertension (*n* = 8), dyslipidaemia (*n* = 2) and diabetes (*n* = 8), these were not included in the multivariate model to avoid overfitting and decreasing sample size.

**Table 2.** Multivariate model of the effect of smoking on MRI lesion resolution.

	Lesion persistence		
	RR	95% CI	*p*-value
Smoking	3.44	2.50–4.73	**<0.001**
Past-smokers	18.71	4.69–74.64	**<0.001**
Current-smokers	26.55	6.61–106.63	**<0.001**
Age at relapse	1.00	0.99–1.02	0.64
Female	0.91	0.60–1.38	0.66
Diagnosis of NMOSD-AQP4	1.94	1.17–3.22	**0.01**
Disease duration at admission	0.99	0.97–1.03	0.97
Time between MRI scans	0.93	0.82–1.06	0.29
Region of relapse			
Myelitis versus brain attack	0.76	0.30–1.88	0.55
Myelitis versus optic neuritis	0.74	0.44–1.26	0.27
Brain attack versus optic neuritis	0.98	0.51–1.90	0.96
CNS lesion location			
Supratentorial versus infratentorial	2.22	0.50–9.81	0.29
Supratentorial versus spinal cord	1.15	0.77–1.72	0.48
Infratentorial versus spinal cord	0.52	0.10–2.60	0.42

MRI: magnetic resonance imaging; RR: risk ratio; CI: confidence interval; NMOSD-AQP4: neuromyelitis optica spectrum disorder with aquaporin-4 positive antibody; CNS: central nervous system.

The risk ratio column represents the relative risk of lesion persistence in the follow-up MRI study for each of the covariates included in the model.

The bold values correspond to statistically significant values.

### Association of lesion resolution with relapse recovery

The presence of MRI lesion resolution was significantly associated with better clinical recovery in both NMOSD-AQP4 and MOGAD groups (RR = 1.9, 95% CI = 1.7–2.2, *p* < 0.001). Nearly all patients with partial or complete lesion resolution had some degree of relapse recovery (*n* = 73, 99%). Additionally, most patients with complete resolution on MRI experienced complete clinical relapse recovery (*n* = 34, 83%).

### NMOSD-AQP4 and MOGAD subgroup differences

Compared with MOGAD patients, a lower proportion of patients in the NMOSD-AQP4 subgroup reached complete clinical recovery (*n* = 13, 23% vs *n* = 26, 54%; *p* < 0.001) or complete MRI lesion resolution (*n* = 13, 23% vs *n* = 28, 58%; p < 0.001). The detrimental effect of smoking was observed in both NMOSD-AQP4 and MOGAD patients, with persistent lesions being more frequent in current- and past-smokers compared with never-smokers (NMOSD: RR = 3.3, 95% CI = 2.3–4.7, *p* < 0.001; MOGAD: RR = 5.8, 95% CI = 3.4–9.9, *p* < 0.001). Additionally, the proportion of complete lesion resolution was higher in never-smokers in both NMOSD-AQP4 and MOGAD (Supplemental Table 1).

### MRI lesion resolution across CNS lesion location

In a post hoc analysis, we found that lesion persistence was more common in supratentorial lesions (50%) than in infratentorial lesions (11%) or spinal cord lesions (25%). Supratentorial lesions were associated with a higher risk of lesion persistence compared with infratentorial lesions (RR = 4.5, 95% CI = 1.2–17.6, *p* = 0.03) and spinal cord lesions (RR = 2.0, 95% CI = 1.1–3.6, *p* = 0.017). However, after correction for covariates, there was no significant relationship between CNS lesion location and persistence of lesions in MRI. Additionally, the effect of smoking on lesion persistence was independent of lesion location in the multivariate regression model explained above (Table 2).

## Discussion

This is the first study describing the effect of smoking on imaging resolution of lesions in NMOSD-AQP4 and MOGAD. Smoking was consistently associated with worse MRI lesion resolution, and both current- and past-smokers had significantly increased risk of MRI lesion persistence. Additionally, there were higher proportions of partial lesion resolution in past-smokers compared with current-smokers, and none of the patients in the current-smoker group achieved complete lesion resolution.

Previous studies suggested the association of smoking with higher number of relapses and increased severity of attacks in NMOSD.^9,14^ Also, smoking may impact clinical outcomes in NMOSD-AQP4 due to reducing relapse recovery rather than increasing relapse frequency.^10^ In MOGAD patients, there was no significant correlation of smoking with clinical outcomes, which was likely associated with the lower disability accrual of single relapses in MOGAD.^3,10^ In multiple sclerosis (MS), smoking appears to be associated with increased disability,^15,16^ a higher number of juxta-cortical lesions and of Dawson’s fingers in MRI.^6^ In general, there seems to be a consistent detrimental effect of smoking in neuroinflammatory disorders, possibly mediated by an increased inflammatory activity,^6,14,15^ and/or, in NMOSD-AQP4, by poor tissue damage recovery after relapses.^10^ Smoking increases the release of pro-inflammatory cytokines and activation of the complement pathway,^17–19^ which may enhance tissue damage and hinder recovery after the initial inflammatory response is triggered.

MRI lesion resolution in inflammatory CNS diseases has not been extensively studied, and its impact in clinical outcomes in NMOSD-AQP4 and MOGAD is unclear. A recent study reported better lesion resolution in MOGAD compared with NMOSD-AQP4 or MS, which was not associated with comorbidities, or clinical recovery, but smoking status was not included.^5^ We found a previously unreported association between MRI lesion resolution and clinical recovery after a relapse in NMOSD-AQP4 and MOGAD, which could highlight the relevance of imaging as a biomarker of tissue damage and recovery.

There was worse resolution of lesions in the supratentorial compartment compared with the spinal cord and infratentorial lesions, which is consistent with previous reports of higher reversibility of lesions in the infratentorial compartment in NMOSD-AQP4^20–22^ and spinal cord in MOGAD.^5^ The higher frequency of lesion resolution in these lesions could be related to the greater predominance of non-destructive inflammatory lesions in these CNS locations.^21^ However, we did not find an effect of lesion location in the multivariate analysis, suggesting that the smoking effect on lesion resolution is independent of CNS lesion location.

Our study has several limitations. First, there was limited access to detailed smoking information due to the retrospective design of our study. This may have reduced our power to detect differences between past- and current-smoker groups. Additionally, smoking was self-reported by patients which could influence effect in past-smokers, as patients may not report ongoing smoking habits. Despite these limitations, we were able to detect a clear correlation of smoking with poor lesion resolution, which supports an underlying strong biological effect. There was also limited information about other risk factors that may be associated with smoking; however, the addition of hypertension, diabetes and dyslipidaemia in the multivariate model, despite the small numbers, did not affect the smoking risk. Finally, because we used clinical scans with heterogeneous scanner fields and protocols, more crude qualitative assessment of MRI images was necessary. To mitigate this, we used a uniform scoring system and tested rater variability, which found an almost perfect inter-rater agreement. The results of this study report a previous undisclosed effect of smoking on the imaging of NMOSD-AQP4 and MOGAD and should be confirmed by a prospective study designed to collect detailed smoking history and perform standardized research MRIs for quantitative assessment of lesion evolution.

## Conclusion

Smoking is associated with worse MRI resolution of inflammatory lesions in patients with NMOSD-AQP4 and MOGAD, which is in turn associated with clinical recovery from acute relapses. Overall, our findings support a detrimental effect of smoking in inflammatory CNS diseases. The role of smoking in exacerbating inflammatory disease, as well as vascular disease, may strengthen educational programmes. Additionally, smoking status may be taken into consideration in the clinical management of these conditions, and understanding the underlying pathogenic processes may help develop novel treatments.

## Supplemental Material

sj-docx-1-msj-10.1177_13524585231188485 – Supplemental material for The effect of smoking on MRI lesion resolution in NMOSD-AQP4 and MOGAD Click here for additional data file. Supplemental material, sj-docx-1-msj-10.1177_13524585231188485 for The effect of smoking on MRI lesion resolution in NMOSD-AQP4 and MOGAD by David Berhanu, Miguel Leal Rato, Silvia Messina, Maria Isabel Leite, Ruth Geraldes and Jacqueline Palace in Multiple Sclerosis Journal
