# Supplementary material for: The effect of smoking on MRI lesion resolution in NMOSD-AQP4 and MOGAD
Source: Mult Scler. 2023 Aug 1;29(10):1250–6. doi: 10.1177/13524585231188485 (PMC10503243; doi:10.1177/13524585231188485)
Supplement: sj-docx-1-msj-10.1177_13524585231188485 – Supplemental material for The effect of smoking on MRI lesion resolution in NMOSD-AQP4 and MOGAD [file sj-docx-1-msj-10.1177_13524585231188485.docx]

**SUPPLEMENTAL MATERIAL**

| **Supplemental Table 1.** | | | | | | | | | | | |
| --- | --- | --- | --- | --- | --- | --- | --- | --- | --- | --- | --- |
| **Lesion resolution in NMOSD and MOGAD cohorts.** | | | | | | | | | | | |
|  | NMOSD-AQP4 | | | | |  | MOGAD | | | | |
|  | Total | Never-smoker | Past-smoker | Current-smoker | *p* |  | Total | Never-smoker | Past  Smoker | Current-smoker | *p* |
|  | n=57 | n=38 | n=14 | n=5 |  |  | n=48 | n=27 | n=12 | n=9 |  |
| Lesion Resolution |  |  |  | <0.001 | |  |  |  |  | <0.001 | |
| Absent | 18 (31%) | 2 (5%) | 12 (86%) | 4 (80%) |  |  | 13 (27%) | 0 (0%) | 4 (33%) | 9 (100%) |  |
| Partial | 26 (46%) | 23 (61%) | 2 (14%) | 1 (20%) |  |  | 7 (15%) | 4 (15%) | 3 (25%) | 0 (0%) |  |
| Complete | 13 (23%) | 13 (34%) | 0 (0%) | 0 (0%) |  |  | 28 (58%) | 23 (85%) | 5 (42%) | 0 (0%) |  |
| Data are presented as n (%) and *p* values are from Fisher’s exact test. MOGAD: myelin oligodendrocyte glycoprotein antibody-associated disease; NMOSD-AQP4: neuromyelitis optica spectrum disorder with aquaporin-4 positive antibody. | | | | | | | | | | | |
